# Effects of Tirzepatide on Low-Density Lipoprotein Cholesterol Levels in Adults: A Systematic Review

**DOI:** 10.7759/cureus.88390

**Published:** 2025-07-20

**Authors:** Isaac Hong, Roberto A Hidalgo Ramos, Sebastián Dufner Krieger, Daniela Secades, Marcelo Ortiz, Luis F Moya Porras, Ana L Piedra Pacheco, Jose E Esquivel

**Affiliations:** 1 Faculty of Medicine, University of Costa Rica, San José, CRI; 2 Department of Endocrinology, Hospital San Juan de Dios, San José, CRI

**Keywords:** adults, diabetes, ldl cholesterol, lipoprotein, systematic review, tirzepatide

## Abstract

Tirzepatide, a dual agonist of the glucose-dependent insulinotropic polypeptide and glucagon-like peptide-1 receptors, is recognized for its effectiveness in regulating glucose levels and promoting weight loss. This systematic review aims to evaluate the current evidence on the impact of tirzepatide on low-density lipoprotein (LDL) cholesterol levels in adult patients (i.e., individuals 18 years and older). A comprehensive search was conducted in the PubMed, EMBASE, and Web of Science databases through June 26, 2025, following the Preferred Reporting Items for Systematic Reviews and Meta-Analyses (PRISMA) guidelines. Eligible studies included randomized controlled trials and observational studies in adults that reported LDL levels before and after receiving tirzepatide. The quality assessment was done using the Risk of Bias 2 tool (RoB 2, Cochrane Collaboration, London, UK) and the Risk of Bias in Non-randomized Studies of Interventions (ROBINS-I). This review found that high doses of tirzepatide were associated with moderate reductions in LDL cholesterol levels, accompanied by a 19% reduction and improvements in LDL particle size. However, the findings revealed heterogeneity, with younger individuals (i.e., those 64 years and younger) exhibiting larger reductions in LDL levels compared to older individuals (i.e., those 65 years and older). Observational studies also reported heterogeneous results, limited by confounding factors and co-interventions. Overall, tirzepatide may decrease LDL cholesterol and improve its lipid profile characteristics, especially at high doses (15 mg) and in individuals with metabolic risk factors; however, variability across studies and short follow-up periods limits the ability to draw definitive conclusions. Further research is needed to establish the role of tirzepatide in controlling LDL levels and reducing cardiovascular risk.

## Introduction and background

Dyslipidemia is a condition characterized by abnormal serum cholesterol levels, which are associated with an increased risk of atherosclerotic cardiovascular disease in these patients. It encompasses an elevation of total and low-density lipoprotein (LDL) cholesterol, triglycerides, and changes in other lipid markers. Several management options exist for this condition, including statins, ezetimibe, and PCSK9 inhibitors. New pharmacotherapies are continually being developed and studied [1].

Tirzepatide is a dual glucose-dependent insulinotropic polypeptide receptor and glucagon-like peptide-1 (GLP-1) receptor co-agonist, which the FDA approved in May 2022 for patients with type 2 diabetes mellitus (T2DM). It is characterized by its effectiveness for glucose control and weight loss. However, in recent times, it has gained popularity for its significant ability to help patients lose weight when treated with this medication [2,3].

The evidence regarding the specific impact of tirzepatide on LDL cholesterol and other lipid markers remains scattered across different trials and studies. This systematic review aims to explore, summarize, and analyze the current evidence regarding the effects of tirzepatide on LDL cholesterol levels in the adult population (i.e., individuals 18 years and older), with or without comorbidities such as obesity, prediabetes, dyslipidemia, or hypertension. By doing so, this systematic review may reveal the potential role of tirzepatide for metabolic management and cardiovascular risk reduction.

## Review

Materials and methods

PICO Framework

The population of interest includes human adults (individuals 18 years and older), either healthy or with mild to moderate comorbidities, such as obesity, T2DM, prediabetes, dyslipidemia, or hypertension. The intervention involves the administration of tirzepatide at any approved dose or formulation. While a comparator is not mandatory, when available, it may include individuals not receiving anti-diabetic or anti-obesity pharmacological treatment or those treated with alternative agents within the same therapeutic class. The primary outcome assessed is the change in LDL cholesterol levels, including absolute values, percentage changes, LDL particle size, or related lipid subfraction parameters.

Eligibility Criteria

Studies published within the last 10 years were considered eligible if they involved human adults, were written in English, and reported LDL levels before and after treatment with tirzepatide or compared LDL outcomes between tirzepatide and other groups. The eligible study designs included randomized controlled trials, prospective and retrospective observational studies, cross-sectional analyses, case-control studies, and within-subject pre-post comparisons. Additionally, studies that combined tirzepatide with minor interventions such as exercise or dietary modifications were also included.

Exclusion Criteria

Studies conducted in animals, the pediatric population (i.e., individuals younger than 18 years), or individuals with severe illnesses or significant comorbidities, such as cancer or those undergoing recent surgical procedures, were excluded. Studies in individuals with T1DM were also excluded. Additionally, studies that included the use of tirzepatide in combination with interventions likely to significantly affect LDL levels, such as other anti-obesity medications (e.g., orlistat, GLP-1 receptor agonists, bupropion), statins, other lipid-lowering therapies (e.g., ezetimibe, fibrates, PCSK9 inhibitors), or bariatric surgery, were excluded if these interventions were part of the study's primary focus, intervention strategy, or explicit patient selection criteria.

Search Strategy

Up to June 26th, 2025, a comprehensive literature search following the Preferred Reporting Items for Systematic reviews and Meta-Analyses (PRISMA) guidelines [4] on PubMed, EMBASE, and Web of Science using the following search keys: for PubMed and Web of Science "("Tirzepatide" OR "Mounjaro" OR "Tirzepatida") AND ("LDL" OR "Low Density Lipoprotein" OR "LDL Cholesterol" OR "Low-Density Lipoprotein Cholesterol")" and EMBASE "('tirzepatide'/exp OR 'tirzepatide':ti,ab,kw OR' mounjaro':ti,ab,kw OR 'tirzepatida':ti,ab,kw) AND ('ldl'/exp OR 'low density lipoprotein':ti,ab,kw OR 'ldl cholesterol':ti,ab,kw OR 'low-density lipoprotein cholesterol':ti,ab,kw)".

Study Selection, Data Extraction, and Quality Assessment

Two authors (Hong, I., and Hidalgo, R.) screened the titles and abstracts of all studies identified in the initial search, which were approved by a third investigator (Ortiz, M.). Studies that initially met the inclusion criteria were selected for full-text screening. These same reviewers analyzed the full texts for eligibility based on predefined criteria. The studies were included if all reviewers agreed. The three authors performed the data extraction mentioned above, and quality assessment was performed and approved by two reviewers (Dufner, S., and Secades, D.). For quality assessment, the Risk of Bias in Non-randomized Studies of Interventions (ROBINS-I) tool was used for observational studies [5], and the Risk of Bias tool (RoB 2, Cochrane Collaboration, London, UK) was used for randomized controlled trials [6].

Endpoints and Definitions

The primary endpoint of this systematic review was the change in LDL cholesterol levels in adults (i.e., individuals 18 years and older) treated with tirzepatide, including both absolute changes in LDL cholesterol levels (mg/dL) and percentage changes from baseline. When studies included additional LDL-related outcomes, such as LDL particle size and subfraction distribution, these were also considered in this review.

LDL cholesterol is considered the amount of cholesterol carried within LDL particles [1] and is measured by standard laboratory assays unless specified otherwise. Although LDL particle size and subfraction distribution offer additional insights for cardiovascular health, these were not included as formal secondary endpoints due to the limited and inconsistent reporting across the studies. No other secondary endpoints were included in this review.

Results

Study Selection

**Figure 1:**
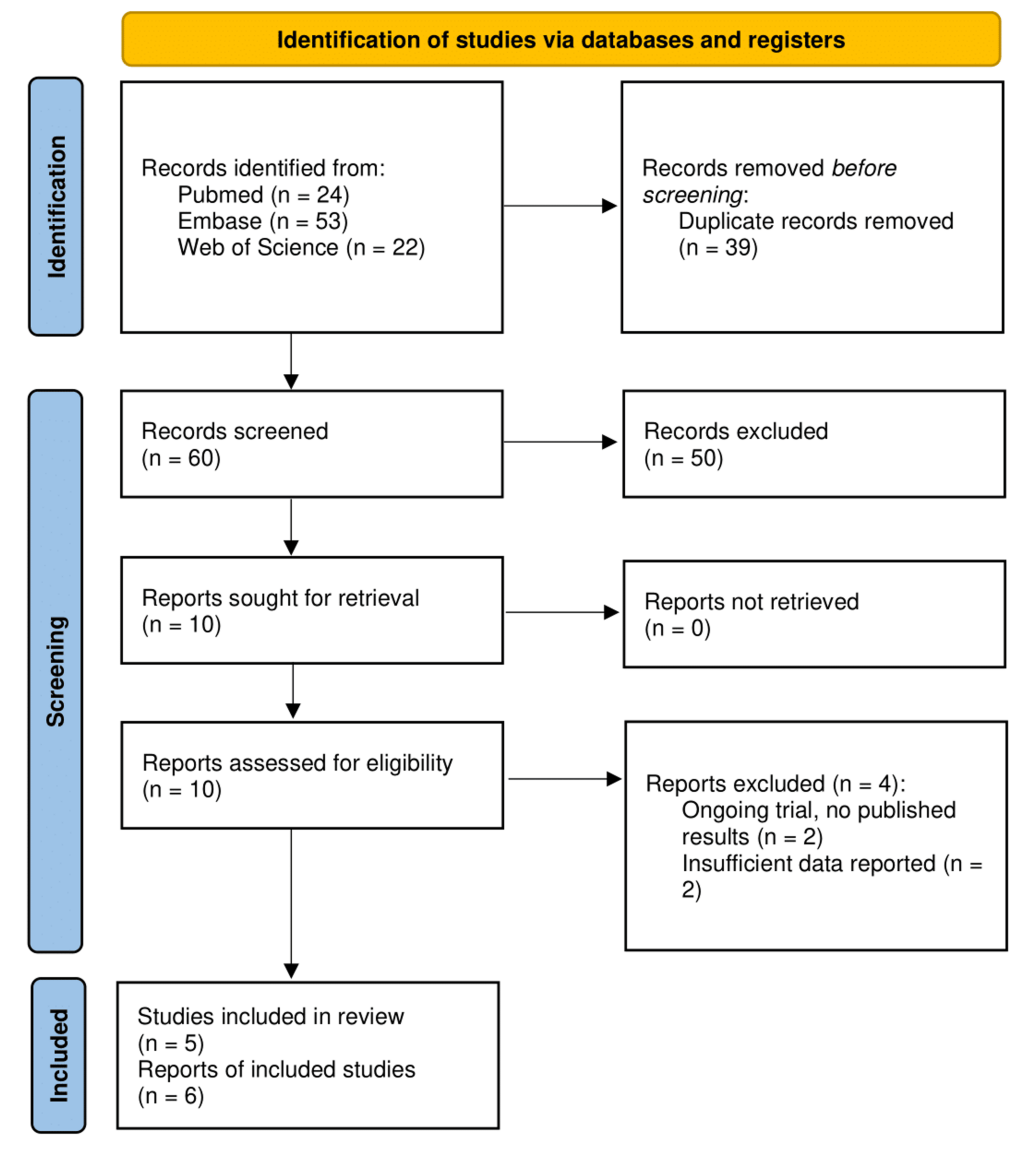
PRISMA flow diagram PRISMA: Preferred Reporting Items for Systematic Reviews and Meta-Analyses

A total of 99 articles were identified across PubMed (n=24), Embase (n=53), and Web of Science (n=22) databases. After removing 39 duplicates, 60 articles were screened by title and abstract, from which 10 were assessed for full-text eligibility. Among the four excluded articles, two were ongoing trials with no published results, and the other two were abstracts with insufficient data reported for data extraction and analysis. Lastly, six articles, comprising five studies and one that branched into two reports, matched the inclusion criteria and had their data extracted for further analysis. This is represented in Figure 1.

Characteristics of Included Studies

This review included five distinct studies, published in six articles, that evaluated the effect of tirzepatide on LDL cholesterol in adult populations. Three randomized, double-blind, placebo-controlled trials [7-9] and three retrospective observational studies conducted in various countries, including Japan, the United States, Iran, and the United Arab Emirates [10-12], were included in this analysis.

All trials were in adult populations, but the specific populations varied considerably. One trial was in young male adults with obesity only [11], while others were in more general populations with T2DM, prediabetes, or metabolic syndrome. Several studies also included participants with other risk factors, such as hypertension or increased waist circumference. Mean or median ages of participants, where reported, varied from approximately 20 to 65 years.

Tirzepatide was administered subcutaneously in doses ranging from 2.5 mg to 15 mg once weekly in all the trials. Tirzepatide was paired with structured exercise programs in a study [7], while in an observational program, the drug was paired with digital health monitoring and lifestyle coaching [12]. The follow-up ranged from six to 26 weeks, depending on the trial.

In one extensive observational study, it was not possible to differentiate LDL findings specifically for the tirzepatide group, as results were combined with those from other anti-obesity drugs [11]. Furthermore, one randomized trial resulted in two separate reports [8,9], each reporting multiple outcome steps or time points, which were both included for a composite evaluation. The table for the characteristics of the studies and their populations is detailed in Table 1.

**Table 1:** Datasheet of included studies BMI: body mass index, CI: confidence interval, COSMOS: clinical obesity strategies monitoring outcomes study, EHR: electronic health record, FDA: Food and Drug Administration, HbA1c: hemoglobin A1c, IDF: International Diabetes Federation, LDL: low-density lipoprotein, LDL-C: low-density lipoprotein cholesterol, NMR: nuclear magnetic resonance, T2DM: type 2 diabetes mellitus

Study ID	Study design	Country/region	Population characteristics	Sample size (tirzepatide group)	Intervention	Comparator	LDL measurement method	LDL outcome (absolute change)	LDL outcome (percent change)	LDL particle size data	Follow-up duration for LDL	Statistical significance reported	Relevant notes/confounding factors
Bagherzadeh-Rahmani et. al., 2024 [7]	Double-blind, placebo-controlled randomized trial	Iran	Adults 20-32 years, BMI >30 kg/m2, fasting blood glucose 100–125 mg/dL, no regular physical activity in the last 6 months, absence of cardiovascular, metabolic, and endocrine diseases, absence of drug use	39	Tirzepatide 2.5 mg or 5 mg subcutaneous, once weekly for 6 weeks. Some groups combined resistance and aerobic training	Placebo and groups with exercise intervention alone or combined with tirzepatide	Standard method	Placebo: 134.1 → 135.2 mg/dL (non-significant). Tirzepatide 2.5 mg: 134.3 → 132.4 mg/dL (p<0.001). Tirzepatide 5 mg: 132.1 → 128.3 mg/dL (p=0.02). Exercise + placebo: 133.6 → 123.4 mg/dL (p<0.001). Exercise + tirzepatide 2.5 mg: 135.7 → 124.1 mg/dL (p<0.001). Exercise + tirzepatide 5 mg: 134.4 → 121.5 mg/dL (p<0.001)	Not reported	Not reported	6 weeks	Yes	Combined interventions with structured exercise, dietary intake controlled. The exercise + placebo group achieved a greater reduction in LDL than the tirzepatide-only group
Frias et. al, 2018 [9]	Randomized, double-blind, placebo-controlled, and active comparator-controlled phase 2 trial	USA, Puerto Rico, Poland, Slovakia	Adults aged 18–75 with T2DM (≥6 months), BMI 23–50 kg/m², HbA1c 7.0–10.5%, on diet/exercise or stable metformin therapy	52 (1 mg), 55 (5 mg), 51 (10 mg), 53 (15 mg)	Tirzepatide once-weekly subcutaneous injection at 1 mg, 5 mg, 10 mg, or 15 mg doses	Placebo and Dulaglutide 1.5 mg once weekly	Laboratory lipid panel (specific method not specified)	No difference in changes in concentrations of LDL between Terzipatide groups vs placebo	Not reported	Not reported	26 weeks	No statistically significant difference in LDL levels	Study funded by Eli Lilly. High attrition, especially in the 15mg group, was mostly due to adverse events
Ishimura et al., 2025 [10]	Retrospective observational pre-post study	Japan	Adults with T2DM. Young = 14 (64 years and younger), elderly = 12 (65 years and older)	26	Tirzepatide 2.5 mg. Route of administration and frequency not specified	No comparator.	Laboratory	Young group: 104.0 ± 27.0 → 87.4 ± 25.1 mg/dL (significant, p=0.006). Elderly group: 106.5 ± 20.4 → 103.1 ± 16.0 mg/dL (not significant, p=0.451)	Not reported	Not reported	3 months	p=0.006 (young group), p=0.451 (elderly group)	78.6% of young patients weighed 80kg or more compared to 36.4% of elderly patients
Ruan et al., 2024 [11]	Retrospective real-world EHR-based observational study	USA	Adults with obesity or overweight. Age range at first diagnosis (37, 63), age median 51	Not isolated	Tirzepatide, real-world dose (not specified)	Other FDA-approved and off-label anti-obesity drugs. Outcomes are analyzed per drug class. Tirzepatide outcomes are extracted separately	EHR measurements	Not reported in values	Tirzepatide demonstrates either significant or visually discernible benefits (i.e., reduction) in LDL levels across all exposure lengths in both cohorts	Not reported	Median treatment session length = UT-physician cohort: 3.5 months; COSMOS cohort: 4.1 months (Short: 30-112 days; Medium: 113-365 days; Long: >365 days)	COSMOS: medium-term LDL reduction (p≤0.01). Short-term LDL reduction (not statistically significant), long-term LDL increase (not statistically significant). All terms LDL reduction (p≤0.05). UT: LDL reduction in medium term and all terms, no statistically significant	Real-world data. Other co-interventions or comorbidities are uncontrolled
Wilson et. al., 2020 [8]	Randomized, double-blind, placebo-controlled phase 2b clinical trial	USA, Puerto Rico, Poland, Slovakia	Adults with T2DM	52 (1 mg), 55 (5 mg), 51 (10 mg), 53 (15 mg)	Tirzepatide once-weekly subcutaneous injection at 1 mg, 5 mg, 10 mg, or 15 mg doses	Placebo and Dulaglutide 1.5 mg once weekly	LDL-C measured via enzymatic methods; LDL particle size and count via NMR spectroscopy	LDL-C reduction at 26 weeks with tirzepatide 15 mg: -19.0% (95% CI: -36.0%, -1.9%; p=0.029) compared to placebo	LDL-C reduction for tirzepatide 15 mg: 19.0% reduction vs placebo	Tirzepatide 10 mg and 15 mg reduced small LDL particles by 23.5% (95% CI: -43.3%, -3.6%; p=0.021) and 32.4% (95% CI−53.7%, −11.1%; p=0.003) vs. placebo. Tirzepatide 5mg, 10mg, and 15mg decreased the total LDL particle concentrations compared with placebo	26 weeks	Significant LDL-C reduction with tirzepatide 15 mg vs placebo (p=0.029); reduction in small LDL particles significant for 10 mg and 15 mg	Post hoc biomarker analysis. Trial funded by Eli Lilly
Zakaria et. al., 2025 [12]	Retrospective observational real-world evidence study	United Arab Emirates	Adults aged 18 years and older, diagnosed with metabolic syndrome based on IDF criteria. Mean age = 47.03	31	Tirzepatide, initial dose of 2.5 mg, gradually increasing to either 5.0 mg, 7.5 mg, or 10 mg by the 3-month mark, and reaching 12.5 mg or 15 mg by 6 months	Semaglutide, initial dose of 0.25 mg, increasing to 1 mg at 3 months and reaching 2 mg at 6 months	Laboratory lipid panel	Decrease in LDL-C by -24.46 (mg/dL), p<0.001	Not reported	Not reported	6 months	Significant reduction in LDL-C levels in the tirzepatide group (p<0.001)	No control group. The intervention program combines pharmacotherapy with digital behavioral intervention, with high digital engagement associated with better outcomes

Summary of Key Findings

Recent evidence suggests that tirzepatide may potentially decrease LDL cholesterol levels among adult patients with obesity, T2DM, and related metabolic disorders; however, this impact has shown heterogeneity across different trials conducted.

Controlled trials have consistently shown the most effectiveness in lowering LDL cholesterol levels, particularly at high doses of tirzepatide (15 mg) [7]. One study showed a 19% reduction in LDL cholesterol with a weekly dose of 15 mg, which was also concurrent with improvements in LDL particle size, suggesting changes in lipid profile that could include more than a decrease in LDL [8]. However, not all trials found statistically significant changes in LDL concentrations, and improvements varied with the methods used. For example, trials using more complex lipid particle analysis methods revealed more subtle changes compared to those using conventional laboratory tests [8,9].

In observational studies, results for LDL levels showed significant heterogeneity. One study reported that younger adults (i.e., individuals 64 years and younger) showed significant decreases in LDL levels after tirzepatide treatment. In contrast, older adults (i.e., individuals 65 years and older) showed little to no improvement [10], suggesting that age or initial metabolic status may influence lipid sensitivity. Additionally, a real-world study combining tirzepatide with digital health support demonstrated improvements in LDL levels; however, due to multiple interventions and aggregated reporting, it is challenging to attribute this effect directly to tirzepatide [12]. Finally, another real-world study demonstrated some benefits in LDL reduction; however, most of the results were not statistically significant, and this study did not account for co-interventions and comorbidities [11].

In summary, tirzepatide appears to have inconsistent effects on LDL cholesterol, particularly at higher doses; however, the results of several studies have some inconsistencies. Differences in study populations, designs, treatment strategies, and assessment methods underscore the need for further studies to establish the role of tirzepatide in managing lipid profiles and reducing cardiovascular risk.

Quality Assessment

**Figure 2:**
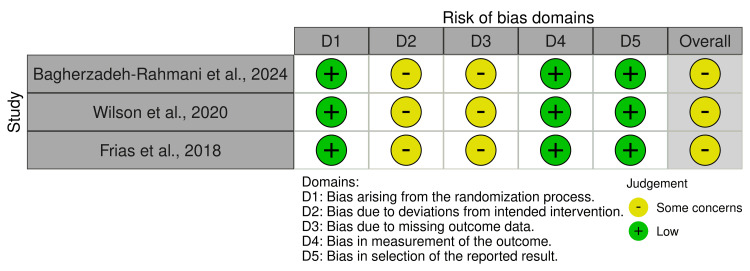
Traffic light plot of included randomized controlled trials The figure illustrates the risk of bias assessment for each of the included randomized controlled trials [7-9].

**Figure 3:**
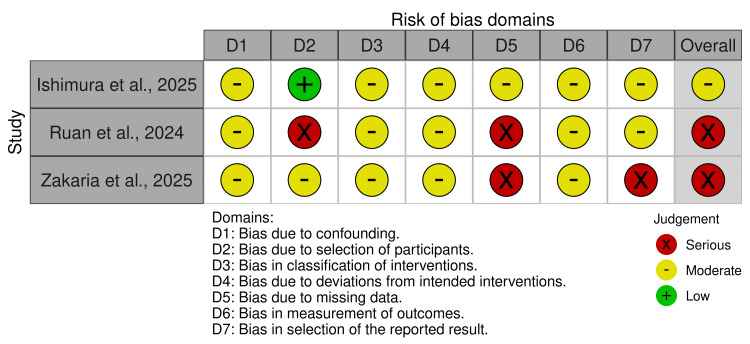
Traffic light plot of included observational studies The figure illustrates the risk of bias assessment for each of the included observational studies [10-12].

The methodological quality of the included studies was evaluated using the RoB 2 tool for randomized controlled studies and the ROBINS-I tool for observational studies. Results are presented in the following tables (Tables 2-3) and traffic light plots (Figures 2-3).

**Table 2:** RoB 2 quality assessment RoBL Risk of Bias

Study	Randomization process	Deviations from intended interventions	Missing data	Measurement of outcome	Selective reporting	Overall risk
Bagherzadeh-Rahmani et al., 2024 [7]	Low risk	Some concerns	Some concerns	Low risk	Low risk	Some concerns
Wilson et al., 2020 [8]	Low risk	Some concerns	Some concerns	Low risk	Low risk	Some concerns
Frias et al., 2018 [9]	Low risk	Some concerns	Some concerns	Low risk	Low risk	Some concerns

**Table 3:** ROBINS-I quality assessment ROBINS-I: Risk of Bias in Non-randomized Studies of Interventions

Study	Confounding	Selection of participants	Classification of interventions	Deviations from intended interventions	Missing data	Measurement of outcome	Selection of reported result	Overall risk
Ishimura et al., 2025 [10]	Moderate risk	Low risk	Moderate risk	Moderate risk	Moderate risk	Moderate risk	Moderate risk	Moderate
Ruan et al., 2024 [11]	Serious risk	Serious risk	Moderate risk	Moderate risk	Serious risk	Moderate risk	Moderate risk	Serious
Zakaria et al., 2025 [12]	Serious risk	Moderate risk	Moderate risk	Moderate risk	Serious risk	Moderate risk	Serious risk	Serious

Each of the randomized controlled trials was rated as having "some concerns" overall. Although randomization processes were generally considered appropriate, and the outcome measures were identified as reliable, concerns were raised about missing outcome data, particularly in the higher-dose tirzepatide groups, as well as potential deviations from the intended interventions. In one study, the combination of tirzepatide with a formal exercise routine introduced a further level of complexity [7], which could potentially have influenced the independent assessment of drug effects.

In the observational studies, heterogeneity in risk of bias was high. In one specific real-world study, the overall risk was moderate, which was primarily contributed to by inadequate control of confounding factors and missing data [10]. The other two observational studies had a high risk of bias due to factors such as inadequate adjustment for confounders, potential selection bias, and inadequate reporting of important outcomes [11,12].

In summary, the randomized trials represented the most valid evidence; however, the missing data and the co-interventions should be taken into consideration when assessing the results. Meanwhile, the observational studies contained valuable real-world information but were accompanied by larger methodological differences, which highlights the need for careful attention when assessing their results.

Discussion

The objective of this systematic review was to investigate the current evidence about the effect of tirzepatide on LDL cholesterol in adult populations. The findings suggest that tirzepatide may have a positive effect on LDL cholesterol; however, they also reveal significant heterogeneity among studies, populations, and intervention strategies. A meta-analysis was not conducted due to the substantial variability in study methodology and reporting, which limited the possibility of quantitatively pooling the results.

Randomized controlled trials have shown modest reductions in LDL cholesterol levels when larger doses of tirzepatide are used. In one such study, a 15 mg dose of tirzepatide resulted in a 19% reduction in LDL cholesterol levels, accompanied by improvements in LDL particle size, indicating both quantitative and qualitative changes in the lipid profile [8]. The improvement in LDL particle size is particularly relevant, as smaller, denser LDL particles are more strongly associated with an increased risk of atherosclerotic cardiovascular disease compared to their counterparts [13].

The lipid-lowering effects observed with tirzepatide are consistent with its mechanism of action as a dual agonist of the GIP and GLP-1 receptors. In addition to regulating glucose metabolism and promoting weight loss, tirzepatide has been shown to reduce hepatic fat accumulation, which may enhance lipid handling by the liver and thus contribute to lower circulating LDL cholesterol levels. The improvements in insulin sensitivity and changes in body composition may also influence lipid metabolism. Emerging evidence suggests that GIP receptor activation, in addition to GLP-1 agonism, may offer additive benefits in modulating lipoprotein profiles. These mechanisms may explain the decreases in LDL seen in patients with tirzepatide therapy [14].

However, not every clinical trial showed statistically significant changes in LDL levels, highlighting ongoing uncertainties in this area. In particular, the use of standard laboratory lipid panels was unsuccessful in detecting subtle changes, which could potentially be achieved through more advanced lipid particle analyses [9]. This disparity underscores the importance of future research employing standardized and comprehensive lipid profiling methods when assessing metabolic interventions, such as tirzepatide. Observational trials provided further insight but showed more variability in LDL responses. In young adults with T2DM, tirzepatide therapy was found to cause marked decreases in LDL levels, compared with minor responses seen in older adults [10]. Such age-associated disparity could be explained by different metabolic processes between the two groups, including altered lipid metabolism and blunted drug responsiveness [15].

Additionally, a randomized controlled trial that combined tirzepatide with lifestyle changes and computerized health support showed an overall improvement in LDL levels; however, due to the collective reporting methodology and the occurrence of co-interventions, it was not possible to separate the effect of tirzepatide [12]. This is more of a concern with real-world studies, as their value lies in providing context, but they commonly introduce confounders that affect outcomes.

In particular, one study examined the combined action of tirzepatide alongside supervised exercise. While both interventions had independent effects regarding LDL level reductions, their combination did not result in an additive effect beyond the benefits of exercise alone [7]. This observation suggests either a possible ceiling effect with lifestyle interventions or the involvement of overlapping pathways controlling lipid metabolism.

Despite the promising trends, several limitations must be noted. Across all studies, there was marked heterogeneity in design, population demographics, intervention regimens, and outcome measurements. Most trials lasted for a limited time and were thus restricted in making inferences about the long-term sustainability of LDL changes or their effects on cardiovascular risk reduction.

Additionally, the quality assessment revealed considerable methodological flaws, particularly in the observational studies. The identified problems included inadequate adjustment for confounding variables, missing data, and selection bias, underscoring the need for cautious interpretation of such findings.

It is essential to consider the clinical relevance of the observed LDL effects. While small decreases in LDL are beneficial, the effect of tirzepatide on long-term cardiovascular outcomes, based solely on its potential effect on LDL, remains uncertain. Currently, most cardiovascular benefits attributed to GLP-1-based drugs, such as tirzepatide, are believed to be due to their antihyperglycemic activity, weight loss, and anti-inflammatory effects; however, lipid-modifying effects require further evidence [14].

The overall results suggest that tirzepatide can deliver greater metabolic effects by optimizing LDL cholesterol levels, especially in high-risk populations and at high dosages. However, more comprehensive and longer-term clinical studies are required to confirm these results, develop standardized measure parameters, and identify the potential role of tirzepatide in lipid profile management and the prevention of cardiovascular risk.

Strengths and limitations

This review's strengths include adherence to PRISMA guidelines and a rigorous search strategy to identify studies focused on tirzepatide and LDL cholesterol. However, limitations include heterogeneity in the studies' designs, measurement techniques, and the frequent use of co-interventions, which limits the ability to attribute effects solely to tirzepatide. Additionally, the limited data on LDL subfraction parameters limit a more comprehensive analysis of lipid risk.

## Conclusions

This systematic review suggests that tirzepatide may contribute to reductions in LDL cholesterol levels, potentially influenced by dosage and patient characteristics. Its mechanisms, including weight loss, improved insulin sensitivity, and hepatic lipid regulation, support a possible role in lipid management. However, due to methodological differences, short follow-up durations, and frequent co-interventions across the included studies, definitive conclusions about the isolated effect of tirzepatide on LDL cholesterol cannot yet be drawn. Further studies with standardized lipid measurements and longer follow-up durations are needed to assess the role of tirzepatide in LDL management and its potential for reducing cardiovascular risk in adults.
